# BICORN (Bilateral Iatrogenic Coronary Obstruction Risk Nullification) Procedure for Transcatheter Aortic Valve-in-Valve Replacement

**DOI:** 10.1016/j.jaccas.2025.105145

**Published:** 2025-09-10

**Authors:** Tariq Ahmad, Vernon Mascarenhas, Terry Bauch, Tyler Wallen, Russell Allan Carter

**Affiliations:** Richard & Marion Pearsall Heart Hospital, Geisinger Health System, Wilkes-Barre, Pennsylvania, USA

**Keywords:** hemodynamics, inotropes, insufficiency, left ventricle, myocardial ischemia, reduced ejection fraction, systolic heart failure, valve replacement

## Abstract

**Objectives:**

We describe a bilateral coronary leaflet modification technique that involves translocation of degenerated transcatheter valve leaflets before placement of valve-in-valve, named Bilateral Iatrogenic Coronary Obstruction Risk Nullification (BICORN).

**Key Steps:**

Key steps include the following: 1) Electrified 0.014-in ASTATO wires were traversed through the base of the right and left coronary leaflets of preexisting CoreValve, which were then swapped with 0.035-in wires; 2) Using peripheral balloons, leaflet traversal points were appropriately dilated; 3) A 26-mm Sapien valve was then advanced over the wire through the left coronary leaflet to the ascending aorta in a deployment-ready state; and 4) Right coronary leaflet was then translocated with 18-mm True balloon followed by deployment of the Sapien valve lined up to sixth node of the CoreValve.

**Potential Pitfalls:**

Translocation of preexisting valve leaflets is an uncontrolled step and can potentially lead to distal embolization and or severe aortic valve insufficiency.

**Take-Home Messages:**

BICORN can be considered as an option for transcatheter aortic valve in transcatheter aortic valve replacement in patients with extreme surgical risk.

## Visual Summary

**Figure:**
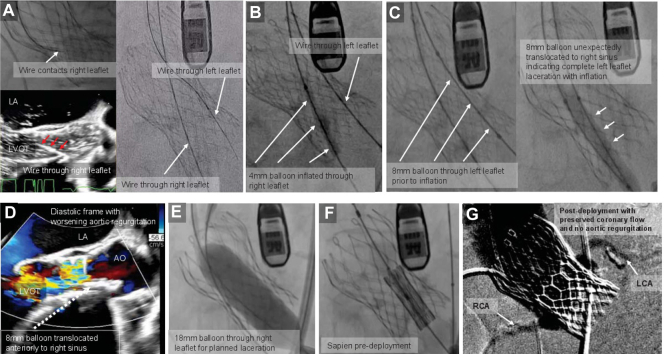

Iatrogenic coronary obstruction can be a life-threatening complication of transcatheter aortic valve replacement (TAVR),^1^ with an increased incidence for valve-in-valve procedures. Bioprosthetic or native Aortic Scallop Intentional Laceration to prevent Iatrogenic Coronary Artery obstruction (BASILICA) or UNdermining Iatrogenic Coronary Obstruction with Radio frequency Needle (UNICORN) are effective leaflet modification techniques, described previously to mitigate the risk of potentially fatal coronary obstruction.^2^^,^^3^ The volume of valve-in-valve TAVR cases is expected to increase given the expanding indications for TAVR. Currently available transcatheter aortic valves have a circumferential nitinol cage in addition to their leaflets, which can create challenges for leaflet modification techniques. The BASILICA technique can fail to adequately splay the lacerated leaflet when the leaflet abuts the cage, or cause leaflet embolization in cases of complete leaflet-prosthesis separation. The UNICORN technique adds simultaneous trapping of the lacerated leaflet away from the coronary ostium, which avoids leaflet embolization in cases of complete leaflet-prosthesis separation. However, the UNICORN technique has not been reported in cases requiring modification of both right and left coronary leaflets. Valve-in-valve cases which require modification of both leaflets carry the additional risk of acute hemodynamic compromise because of the associated acute severe aortic regurgitation postlaceration. Our team elected to apply the UNICORN method in a case requiring dual-leaflet modification, and we report this first experience of Bilateral Iatrogenic Coronary Obstruction Risk Nullification (BICORN) in a patient with degenerative bioprosthetic CoreValve failure with severe insufficiency leading to refractory shock necessitating a transcatheter aortic valve in TAVR procedure.

**Take-Home Messages**

• Iatrogenic coronary obstruction can be a life-threatening complication of transcatheter aortic valve replacement, especially posing a bigger challenge for valve-in-valve procedures.
• BICORN can be considered as an option for transcatheter aortic valve in TAVR procedure in extreme surgical risk patients.
• Balloon-assisted leaflet translocation cannot be fully controlled and can potentially lead to coronary obstruction including leaflet embolization.
• Further bench testing will be required to assess the reproducibility of this procedure.

## Case Summary

**Figure 1:**
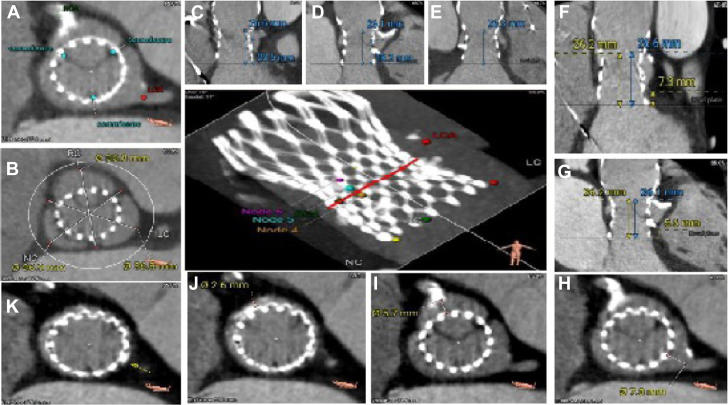
Computed Tomography Transcatheter Aortic Valve Replacement Measurements for Valve-in-Valve Procedure Planning (A) CoreValve commissures in relation to the left and right coronary arteries. (B) Sinus of Valsalva diameters. (C) Left sinus height. (D) Right sinus height. (E) Noncoronary sinus height. (F) Left coronary sinus approximate distances, including CoreValve inflow to native annulus, CoreValve inflow to sinotubular junction (STJ), and CoreValve pinned leaflet height with STJ, are 0.4 mm above the top of the pinned leaflet. (G) Right coronary sinus approximate distances, including CoreValve inflow to native annulus, CoreValve inflow to STJ, and CoreValve pinned leaflet height with STJ, are 0.1 mm above the top of the pinned leaflet. (H) Valve to left coronary artery distance. (I) Valve to right coronary artery distance. (J) Valve to STJ in right coronary artery direction. (K) Valve to STJ in left coronary artery direction.

The case was a 75-year-old man with prior trileaflet aortic valve stenosis who underwent TAVR using a 31-mm Medtronic CoreValve. Nine years later, he was readmitted to the hospital with cardiogenic shock requiring inotropic support, new severe left ventricular dilation, and a decreased left ventricular ejection fraction of 20%. Transesophageal echocardiogram revealed severe intravalvular aortic insufficiency (Video 1) without leaflet thickening. Cardiac catheterization showed a calcified proximal 80% dominant right coronary artery stenosis without anginal symptoms (Video 2). Because of extremely elevated surgical risk in a frail patient, computed tomography was completed showing no leaflet thrombosis but low sinotubular junction (STJ) with valve to STJ of 0 mm on the left and 2.6 mm on the right (Figures 1J and 1K) in preparation for a possible TAVR procedure. The CoreValve was noted to be aligned with native aortic valve sinuses. He remained inotrope dependent. Balloon augmented BASILICA of leaflets was considered, but there was concern about inadequate leaflets splay within the preexisting CoreValve frame and hence an increased risk of coronary obstruction. After heart team discussions, the decision was made to proceed with transcatheter aortic valve in TAVR. We planned to use a 26-mm Sapien Ultra Resilia valve lined up at the 6th node of the CoreValve cage cells, to avoid leaflet overhang,^4^ along with leaflet modification of the left coronary leaflet using the UNICORN technique but with an electrified coronary wire instead of a radiofrequency needle, followed by balloon-assisted translocation of the right coronary leaflet.

## Procedural Steps

The procedure was performed under general anesthesia along with intraoperative transesophageal echocardiogram guidance. Bilateral femoral arterial and venous accesses were obtained. A 14-F Gore DrySeal sheath was placed in the right common femoral artery, and a 14-F Edwards sheath was placed in the left common femoral artery. A Judkins-4 guide catheter was placed in the right coronary cusp of the CoreValve. An electrified Astato-30 wire through piggyback converter was then advanced through the base of the leaflet. Next, a Quick Cross catheter was advanced over the piggyback converter in the left ventricle, and the wire was exchanged with an Amplatz Extra Stiff wire. Then, following the same steps but with the use of an Amplatz left-2 guide catheter (Video 3), a second Amplatz Super Stiff wire was placed into the left ventricle through the base of the left coronary cusp of the CoreValve. A 26-mm Sapien Ultra valve was appropriately loaded to be ready. Using a 4-mm balloon, predilation of both leaflets' traversal points was performed. Next, an 18-mm True balloon was placed across the right leaflet, followed by further predilation of left coronary cusp with 8 mm over the wire balloon to facilitate the passage of the Spain valve through it. The 8-mm balloon was removed, and the Sapien Ultra valve was advanced to the thoracic aorta and loaded on the balloon. The valve was then parked in the ascending aorta. No significant change in systemic pressure was noted at this point. Next, laceration of the right leaflet was successfully performed with dilation of an 18-mm True balloon (Video 3), which was then quickly removed along with the wire. Severe valvular insufficiency was noted (Video 3) with systemic pressure drop by 10 mm Hg systolic (baseline 100 mm Hg) along with rise of heart rate by >30 beats/min (baseline: 92 beats/min) immediately. The Sapien valve was then quickly advanced and deployed at the intended depth under rapid pacing (Video 3). Postdeployment, systemic pressures were noted to rise to 170/90 mm Hg. Brisk bilateral coronary flow was noted on root angiogram (Video 3). Transesophageal echocardiogram showed patent flow in the left main coronary artery and stable left ventricular wall motion. No paravalvular leak was noted with a mean aortic valve gradient of 2.5 mm Hg. Right heart catheterization was performed showing mean pulmonary capillary pressure of 33 mm Hg with a mean right atrial pressure of 16 mm Hg. An intra-aortic balloon pump was placed via the left femoral arterial access which was then removed on postoperative day 1. Ionotropic support was gradually reduced and finally turned off with initiation of guideline-directed medical therapy. Six days later, his pacemaker was upgraded with dual chamber leads including a His bundle pacing lead.

## Potential Pitfalls

The following are the possible important considerations for procedural planning.

1. We planned a focal balloon laceration of the left coronary leaflet to prepare for rapid TAVR deployment through this leaflet immediately after the laceration of the right coronary leaflet. The left leaflet laceration was larger than planned, extending to the tip and creating more aortic regurgitation than desired. We postulate this resulted from orientation of the 8-mm lacerating balloon at a nonperpendicular angle to the leaflet. In future, rapid pacing could foster more optimal balloon orientation. The subsequent procedural steps were still completed rapidly and successfully.
2. Sapien valve deployment at the fifth nodes was discussed but due to possible coronary obstruction at the STJ level with significant STJ calcification in line with coronaries takeoffs and degenerated stiffened CoreValve leaflets along with risk of sinus sequestration with ongoing shock physiology, it was not thought to be a safe option. CoreValve leaflet overhang with Sapien valve deployment at the fourth node was considered by the team to be significant, possibly impacting the long-term functioning of the Sapien valve. Without any form of leaflet modification or with possible inadequate leaflet splay with bilateral BASILICA, deployment at the fifth node was thought to be concerning for sinus sequestration and delayed coronary obstruction with extremely narrow valve to calcified STJ distances. CoreValve to STJ or coronary distance can further decrease with placement of a balloon expandable valve when lined up appropriately to avoid leaflet overhang. This can be more pronounced when more than nominal inflation of the valve is performed, making leaflet modification even more necessary in borderline cases. In our case, coronary obstruction risk was thought to be extremely high without leaflet modifications.
3. Leaflet translocation with balloon expandable valve will possibly trap the preexisting valve leaflet between the cages of both valves, possibly away for the coronary take off because expansion happens from center to outward. In case of leaflet laceration with a balloon followed by valve-in-valve deployment, the lacerated leaflet can fall back in front of the coronary take-off posing a risk of obstruction (Videos 4A and 4B). Also, leaflet laceration is not a fully controlled procedure and potentially can lead to leaflet embolization, leading to significant clinical consequences.
4. Sinus alignment can be more controllable with a self-expanding valve compared with balloon expandable valves. In case of sinus nonalignment, commissural suture line can come in front of the coronary artery causing some degree of obstruction, worse with no valve to coronary distance or with calcific ostial coronary anatomy. On the same lines, the height of the sealing skirt of the transcatheter valve can be important, especially in case of extremely low take off the coronary arteries.
5. In this case, the decision was made against performing percutaneous coronary intervention to the right coronary artery because of ongoing cardiogenic shock, absent anginal symptoms, left ventricular dysfunction being out of proportion to right coronary artery disease, and with preexisting acute renal dysfunction raising the risk of contrast-related renal injury, especially with difficult coronary engagement. Untreated occlusive coronary disease, however, can become clinically more significant even with reduced flow into sinuses post-transcatheter aortic valve in TAVR.

## Conclusions

Our case highlights that BICORN can be considered as an option for transcatheter aortic valve in TAVR procedure in extreme surgical risk patients. With the expected rise in the need for transcatheter aortic valve in TAVR procedure in the future, we hope to see more similar procedures to better understand the effectiveness and safety of this procedure.

**Visual Summary:**
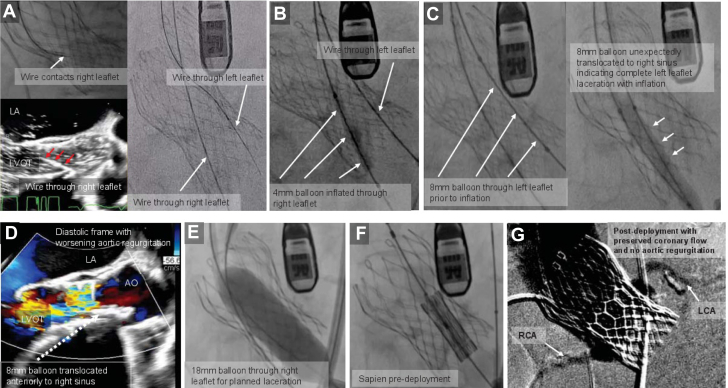
Major Steps in a Due Order for BICORN Procedure

## Funding Support and Author Disclosures

The authors have reported that they have no relationships relevant to the contents of this paper to disclose.

## Appendix

Video 1 Transesophageal Echocardiogram Showing Baseline Aortic Valve Insufficiency

Video 2 Right Coronary Artery Angiogram Showing Proximal Calcified Occlusive Disease

Video 3 Step-by-Step Bilateral Iatrogenic Coronary Obstruction Nullification Procedure for Transcatheter Aortic Valve in Valve Replacement

Video 4 Laceration of CoreValve Leaflet and of the Second Leaflet a) Video demonstrating laceration of CoreValve leaflet with 18 mm balloon displacing the lacerated leaflet away from the valve cage b) laceration of second leaflet showing laterally displaced leaflet partially covering the cells of the cage in the position of previously lacerated leaflet in video a.
